# Crosstalk between Tumor-Associated Macrophages and MicroRNAs: A Key Role in Tumor Microenvironment

**DOI:** 10.3390/ijms232113258

**Published:** 2022-10-31

**Authors:** Xianghong Zhou, Bo Chen, Zilong Zhang, Yin Huang, Jinze Li, Qiang Wei, Dehong Cao, Jianzhong Ai

**Affiliations:** Department of Urology, Institute of Urology, Sichuan University, Chengdu 610041, China

**Keywords:** tumor-associated macrophages, microRNAs, tumor microenvironment, crosstalk

## Abstract

As an in-depth understanding of immunotherapy continues to grow, current anticancer therapy research is increasingly focused on the tumor microenvironment (TME). MicroRNAs (miRNAs) play crucial roles in the regulation of genetic information and expression and mediate interactions between tumor cells and components in the TME, such as tumor-associated macrophages (macrophages). Macrophages are abundant in the TME, and their different polarization directions can promote or inhibit tumor growth and progression. By regulating biological behaviors, such as macrophage recruitment, infiltration, and polarization, miRNAs can affect various molecular pathways to regulate tumor progression and treatment response. In this review, we discuss in detail the effects of macrophages on tumors and the multifaceted effects of miRNAs on macrophages. We also discuss the potential clinical applications and prospects of targeted therapy based on miRNAs, novel clinical biomarkers, and drug delivery systems.

## 1. Introduction

The tumor microenvironment (TME) refers to the internal environment of the tumor and its role in the origin and survival of tumor cells. The TME includes cancer and immune cells, microvascular components, and various cytokines as well as the extracellular matrix [1]. The TME is a complex integrated system that is a “soil” for tumors and their growth. The TME affects the production and progress of tumors. Tumors can promote the formation and immune tolerance of tumor blood vessels to affect the TME by releasing signal factors [2]. Immune cells and other TME components can affect the growth and development of cancer cells. Influential immune cells, such as regulating T cells (Tregs), participate in the formation of the TME to induce the production of TME inflammation and promote immune escape and malignant development [3,4]. Many other components also play an important role in the interaction between TME and tumors. For example, cancer-associated fibroblasts can release matrix cell derivatives and vascular-generating factors. Vascular endothelial cells mainly regulate the tumor vascular production, which are all important foundations for promoting tumor cell growth and metastasis [5]. Understanding the interaction between the cells in TME is essential for cancer treatment and can better guide the precision medical treatment of tumors.

Immunotherapy has provided innovative treatment options for many kinds of tumors and can help improve the clinical outcomes of patients. However, there are substantial problems with poor treatment responses [6]. Currently approved immunotherapy depends on the immune checkpoint inhibitors or the administration of transformed T cells in the patient to enhance the T cell function [7,8]. In addition to T cells, other immune cells in the TME can also affect tumors [9]. Macrophages are usually the richest immune cells in the TME except for T cells. Therefore, there is considerable interest focused on understanding the interaction between macrophages and tumor cells to overcome the limitations of current immunotherapy and search for novel important biomarkers [9]. Macrophages exist in almost all tissues and organs of the body and participate in congenital and adaptive immunity, which is essential for maintaining physiological conditions [10]. Macrophages can rapidly respond to foreign organisms, antigens, and toxins. Macrophages in the TME are called tumor-associated macrophages (macrophages) and have a variety of sources and functions. The existence of high numbers of macrophages near tumor cells shows that they participate in the formation of TME, although their specific impact on the occurrence and development of tumors still needs to be studied in-depth. Accumulating evidence indicates that macrophages have a complex effect on various cancers. Macrophages are known to have a two-sided response to the local environmental signals and can express inflammatory or anti-inflammatory effects as well as the influence of tumor or antitumor treatments [10,11].

MicroRNAs (miRNAs) are a non-coding single-chain small RNA of approximately 22–24 nucleotides and are highly conserved in evolution and exist widely in cells [12]. miRNA biology has been greatly expanded in more than two decades since the first miRNA was discovered. miRNAs play a vital regulatory role in cells, especially in messenger RNA (mRNA) post-transcriptional regulation, through the combination of the mRNA 3′-untranslated region (3′-UTR) to regulate mRNA expression, inducing inhibition of translation efficiency and instability of the mRNA [13]. This miRNA-driven mRNA transcription can be adjusted to regulate the characteristics of various cancers, such as their occurrence, development, metastasis, and therapeutic resistance. Therefore, it is necessary to understand the important role of the complex interactions between cancer and immune cells [14]. In this review, we discuss the latest in this field and focus on the important impact of macrophages in the occurrence and progression of tumors, as well as the role played by miRNAs in the regulation of macrophages in TME.

## 2. Tumor-Associated Macrophages

### 2.1. Origin of Tumor-Associated Macrophages

Macrophages exist almost everywhere in the body and are essential for maintaining normal bodily functions. According to the difference in origin, macrophages can be initially divided into tissue-resident or monocyte-derived macrophages [15,16]. Tissue-resident macrophages are derived from hematopoietic ancestral cells, usually settled in targeted tissues. They are known under various names, such as microglia in the brain, Langerhans cells in the epidermis, Kuffer cells in the liver, and macrophages in the alveoli [17,18]. Tissue-resident macrophages are the earliest immune cells that interact with cancer cells during tumor formation and are known to be important in the development of tumor growth and metastasis in cancer [19]. 

The monocyte-derived macrophages that originate in the circulating monocytes of the periphery are considered classical macrophages [16,20]. After tumor formation, the recruitment of monocytes is related to various regulatory factors in the TME, including growth factors, chemokines, and cytokines [20]. When monocytes migrate to tumor tissue, they quickly differentiate into macrophages in the TME. This differentiation process is closely related to conditions, such as local hypoxia in TME [21,22]. Concurrently, factors, such as interleukin (IL)-10 secreted by cancer or interstitial cells, are also important in the differentiation from monocytes to macrophages rather than to dendritic cells [22]. Macrophages derived from monocytes then stimulate the progression and survival of tumor cells through various signal pathways [23].

The participation of tissue-resident macrophages for normal homeostasis differs among different tissue. There are a few examples. For instance, Kuffer cells are the tissue-resident macrophages of the liver that display high phagocytic and lysosomal activity because of their specialized role in monitoring and filtering blood entering the liver. Kuffer cells form a protective barrier against systemic pathogen circulation and remove waste red blood cells [24]. Kuffer cells also perform liver-specific metabolic functions and regulate plasma cholesterol levels. However, under disease conditions, they can be pathologically activated and can then cause hepatocyte damage [25]. In the lung, alveolar macrophages interact with alveolar epithelial cells via CD200- and transforming growth factor (TGF-β) signaling pathways to maintain their non-inflammatory state [26,27]. Alveolar macrophages also excel in bacterial phagocytosis and help in resistance to viral infections in the lung [28]. Depletion of alveolar macrophages prior to bacterial or viral infection not only reduces pathogen clearance but also increases inflammation [29]. Microglia are tissue-resident macrophages of the central nervous system that play a major role in homeostasis maintenance and immune defense in the adult central nervous system [30]. Microglia are involved in physiological and biochemical activities to functionally support the blood–brain barrier and make an important contribution to the specific immune state of the central nervous system.

Similarly, the role of both types of macrophages in cancer development is thought to be organ specific. In breast cancer models, the number of tissue-resident macrophages decreases over time, whereas the number of monocyte-derived macrophages simultaneously increases [31]. In this case, ablation of tissue-resident macrophages had less effect on tumor growth, whereas ablation of monocyte-derived macrophages decrease tumor size. In contrast, in the pancreatic cancer model, tissue-resident macrophages increase during tumor progression and acquire a transcriptional profile favoring a profibrotic program for pancreatic cancer growth. This program is not disrupted by the depletion of monocyte-derived macrophages but is reversed mainly by tissue-resident macrophage depletion [32]. In lung cancer models, macrophages of both sources were found to contribute to tumor proliferation and enhanced invasiveness [19].

### 2.2. Polarization of Tumor-Associated Macrophages: Double-Edged Sword

Macrophages are a key participant in tumor-related immunity because they can support or inhibit tumor growth based on their phenotype and function. These phenotypes and functions are diverse, and they can affect the effect of almost any type of anticancer treatment [33]. Therefore, it is particularly important to understand the complexity of macrophages and their regulation mechanism to interact with tumor cells. Macrophages are highly plastic in terms of polarization and exert different functions according to local conditions [34]. Non-polarized macrophages are called M0 macrophages, which can be polarized to M1 (classically activated macrophages) and M2 (replacement activated macrophages) types. Furthermore, M1 or M2 type macrophages can also be re-programmed to the opposing activated form [35,36].

The differences between the local cytokine environments and induction agents are the main factors that produce different polarization directions for macrophages [35,37]. M1 and M2 polarization are significant features for macrophages to participate in many biological processes. M1 macrophages are called classic macrophages that can be activated by bacterial products or interferon-γ (IFN-γ). M1 macrophages can secret high levels of inflammatory cytokines and inhibitory factors related to antitumor immunity, including tumor necrosis factor (TNF)-α, IL-6, IL-12, and C-X-C motif chemokine ligand (CXCL) 10. M1 macrophages have a powerful antibacterial function and play a significant role in inhibiting tumor growth [38,39]. In M2 macrophages, immune-regulating cytokines, such as IL-4, IL-10, and TGF-β, can induce M2 macrophage polarization [39,40]. IL-4 receptor-α (IL-4R-α) can activate signal transducer and transcription activation agent 6 (STAT6) [41,42]. Th2 cytokine IL-4/IL-13 induced the polarization of M2 macrophages. M2 macrophages are known to have anti-inflammatory and powerful phagocytotic capabilities that can eliminate apoptotic cells, treat chronic infections, and promote wound healing. M2 macrophages also secrete tissue reshaping and vascular generating factors, such as vascular endothelial growth factor (VEGF), related to tumor promotion. Different cytokines are the key biomarkers of polarized macrophages. M1 macrophages are usually related to IL-12, whereas M2 macrophages are related to IL-10 [43,44]. In addition, M2 macrophages can be divided into four different subtypes: M2a, M2b, M2c, and M2d [45,46]. M2a macrophages are induced by IL-4 and IL-13, and express high levels of IL-R and C-C motif chemokine ligand 17 (CCL17). They secrete fibrous factors, such as TGF-β, insulin-like growth factor, and fibrin to promote tissue repair [23,47]. M2b macrophages, known as regulatory macrophages, have high levels of CCL1 and TNF superfamily members 14 (TNFSF14) [45,48]. M2b macrophages can regulate the immune and inflammatory responses. They are believed to promote tumor development and infections with parasites, bacteria, and fungi by weakening immunity and inflammatory responses [23,49]. M2c macrophages are often referred to as acquired macrophages and release a large amount of IL-10 by secreting high levels of TGF-β and promoting fibrosis activity resulting in a strong anti-inflammatory activity. In addition, M2c macrophages show highly expressed levels of Mer receptor tyrosine kinase, which decreases the effective devouring effect [50]. M2d macrophages are induced through Toll-like receptors (TLR) ligands and A2 adenosine receptors (A2R) agonists or IL-6 co-stimulation [35]. The main features of these cells are the generation of high levels of IL-10, TGF-β, and VEGF and low levels of IL-12, TNF-α, and IL-1β that help generate vascular production and cancer metastasis [35,51]. 

In the TME, macrophages mainly exercise tumor-related biological functions in the form of TAMs. TAMs are activated by factors secreted by tumors, and their phenotype depends on the type of tumor entity, and stage of tumor development (primary tumor or metastasis) [52,53]. The traditional M1/M2 phenotype can partially distinguish the difference in tumor biological functions of TAMs. In the early stages of cancer, the M1 macrophages are more abundant, but as cancer cells hijack the metabolism, the proportion of M2 macrophages increases dramatically [46]. M1 macrophages are the main immune cells that inhibit tumors, and M2 macrophages can act to promote the progression of tumors. In addition, in a special TME, polarized macrophages usually interact with other immune cells, such as natural killing cells, CD8+ T cells, and Treg cells, and can affect further tumor suppression or progress [54,55]. Many studies have found that high-level enrichment of M1 macrophages in the center of tumor tissues foreshadows improved survival for patients with cancer, whereas M2 macrophage enrichment predicts poor prognosis [56,57]. Many types of cancer are related to the M2 polarity of macrophages. The emerging treatment strategies for macrophages are mostly targeted at M2 macrophages. By suppressing or activating circulating monocytes in the TME, the generation of macrophages can be eliminated or their polarization from M2 to M1 regulated or revised to remove them [58,59]. The plasticity and diversity of macrophages partly help to explain their different functions, although the complexity of macrophages cannot be easily explained by this dual classification. In fact, TAMs rarely show a fully defined M1 or M2 phenotype, and several researchers have attempted to classify macrophage populations into other classes (e.g., M2a, M2b, and M2c) or use looser terms (e.g., M1-like and M2-like) [60,61,62]. For example, different subtypes of macrophages were identified that express the characteristics of M1- or M2-related genes, although several tumor-promoting macrophages have been identified with a genetic expression other than the typical genes related to M2 activation [63,64]. For instance, TAMs can exhibit an intermediate M1/M2 state. In addition, in renal cell carcinoma, several markers of M1 and M2 macrophages can be found simultaneously in TAMs isolated from patients, suggesting that several TAMs may exhibit mixed phenotypes [65]. Furthermore, although M1 TAMs are thought to be associated with improved tumor prognosis, this is controversial in some tumors. Melanoma-based studies have found that M1 macrophages are less abundant than M2 macrophages in the early stages of MM, whereas the proportion of the M2 population increases rapidly during MM progression. However, elevated numbers of both M1 and M2 macrophages were associated with poor prognostic indicators and patient survival [66]. Another melanoma study found that M1 macrophages may also contribute to tumor progression by forming a proinflammatory microenvironment [67].

### 2.3. New Research Techniques Bring New Insights into the Complex Role of Tumor-Associated Macrophages

There has recently been a gradual re-evaluation of the molecular characteristics of macrophages based on single-cell sequencing (scRNA-seq), which can deepen our understanding of macrophage diversity and its complex interactions with tumor cells. scRNA-seq analyses of a variety of clinical tumor samples have identified a wider range of macrophage markers to determine a tumor inhibitory or promoting status, outside of the traditional M1/M2 macrophage polarization classification [68]. High expression of matrix metallopeptidase 12 (MMP12) that differs from that found in either M1 or M2 cells was found to be associated with poor clinical outcomes [69]. Although macrophages expressing FABP5 are not identified as M2 macrophages by the traditional polarization term, this type of macrophage was found to exist in invasive cancer TME, producing various immune regulatory molecules (including PD-L1 and PD-L2), and was associated with unfavorable survival rate [70]. Other scRNA-seq studies have also shown that favorable macrophages can increase the expression of genes that may weaken the antitumor immune response at the same time. In a study involving renal cell carcinoma patients, the macrophages of those patients who received anti-PD-1 therapy were mainly shifted to M1, and the functions of the protease body and antigen presentation increased [71]. Similarly, neoadjuvant anti-PD-1 immunotherapy in patients with recurrent glioblastoma triggered a CD8^+^ T cell intratumoral response and changes in macrophages, facilitating T cell recruitment and activation [72]; however, high levels of immunosuppressive factors were also expressed simultaneously. The macrophage state that expresses secreted phosphoprotein 1 (SPP1) promotes vascular production of M2-related genes and is identified in a variety of tumor types, including lung, colorectal, and ovarian cancers as well as pancreatic adenocarcinoma [10,63]. Although our knowledge of the diversity of macrophages remains limited, the application of new technologies, such as scRNA-seq, will help to further understand the complexity of these cells.

## 3. Interaction of MicroRNAs with Tumor-Associated Macrophages

Gene regulation at the post-transcriptional level is an important regulatory mechanism. mRNA can be affected by transcriptional activation or repression, determining the final expression of the protein [73,74]. miRNAs are considered one of the most important regulators at the post-transcriptional level and are found in almost all tissues. The main mechanism by which miRNAs function is to bind to Argonaute proteins, which then intercalate into the RNA-induced silencing complex (RISC). Targeted mRNAs are mainly identified by RISC, which recognizes their 3′-UTR [75]. miRNAs have three different mechanisms to repress gene expression, including enhancing mRNA stability, repressing translation, and mediating mRNA cleavage [75,76]. miRNAs can also mediate and regulate the actions of other non-coding RNAs, including long non-coding-RNAs and circular-RNAs [77]. miRNAs usually have tissue-specific functions and, depending on the cancer type, specific miRNAs can function as oncogenic or tumor suppressors [78]. The ability of miRNAs to modulate the magnitude of the innate immune response has been reported, and miRNAs are also involved in immune and inflammatory responses as part of feedback regulatory mechanisms. Evidence suggests that miRNAs have prominent roles as key regulators of macrophage differentiation, infiltration, and activation [79]. Recent studies have also established the role of miRNAs role in regulating macrophage polarization. In the TME, a complex and highly regulated network of miRNAs act as a bridge and enables the interaction between cancer and various immune cells, especially TAMs. 

### 3.1. Roles of miRNAs on the Differentiation, Recruitment, and Infiltration of Macrophages

Macrophages cannot proliferate or survive a long time in the TME and require constant recruitment of macrophage precursors to tumor sites to replenish populations [80]. The differentiation from hematopoietic stem cells to macrophages involves a series of steps [81]. Runt-associated transcription factor 1 (RUNX1) is a key protein in hematopoietic cell development. Enhancement of miR-129 expression can promote granulation and inhibit monocyte production by inhibiting the expression of RUNX1 [82]. The transcription factor PU.1 is important in myeloid differentiation and can also be regulated by RUNX1. PU.1 can regulate the differentiation process by controlling various miRNAs. Among them, the translation of nuclear factor 1 A (NFIA) can be impaired by the upregulation of miR-424 expression by PU.1 in myeloid cells. Downregulation of NFIA expression causes the expression of macrophage colony-stimulating factor receptor (M-CSFR) [83]. M-CSFR can influence the differentiation of monocyte/macrophage cells [84]. miR-223 regulates the differentiation of precursors in the bone marrow into granulocytes or monocytes and has an important role in altering macrophage polarization and activation [85]. Notch signaling regulates miR-148a-3p upon granulocyte-macrophage colony-stimulating factor (GM-CSF) stimulation and could enhance monocyte-to-macrophage differentiation, as Notch signaling is essential for macrophage differentiation and polarization [86].

Monocyte-derived immune cells, such as macrophages and dendritic cells, are attracted by the TME to actively secrete a series of chemokines, including CCL2, CX3CL1, M-CSF, and colony-stimulating factor 1 (CSF1). miR-125b was found to target the chemokines CSF1 and CX3CL1, thereby impairing the recruitment of macrophages [87]. In liver cancer research, miR-26a is thought to suppress the recruitment of macrophages by targeting M-CSF through the PI3K/Akt pathway [88,89]. CCL2-CCR2 signaling is an important way of recruiting circulating monocytes. In the TME, miR-375 is found to be highly expressed in cancer cells and can regulate the expression of CCL2 to promote the recruitment of macrophages. miR-375 can also be phagocytosed by macrophages and targets Tensin 3 (TNS3) and PXN in macrophages to promote macrophage migration and infiltration into the TME [90].

### 3.2. Regulation of miRNAs on the Polarization of TAMs

Various molecules from the TME can stimulate macrophages to exhibit different phenotypes to perform different biological functions [52]. M1- and M2-like phenotypes are general classifications of macrophages, and the M2 phenotype is closely associated with the tumor-promoting role of macrophages in general. Notably, the classification of macrophage subtypes is complex, and it is difficult to identify specific types of macrophages using a single set of specific markers, so the M1/M2 phenotype classification is only a brief summary. Signaling in the TME is one of the major determinants of macrophage activation and polarization, and factors such as STAT-1, NF-κB, AKT2, TNF-α, TLR, IL-1, and IL-6 are required to maintain the M1 phenotype, whereas IL-12, CCL2, and CXCL10, as well as pathways and molecules, including STAT-6, IL-4R, IRF-4, PPARδ, PPARγ, and JMJD3, are required to maintain the M2 phenotype [91]. These signaling pathways are regulated by a series of miRNAs that indirectly exert antitumor or tumor-suppressive effects through the functional differences of polarized macrophages.

Many miRNAs have been found to promote the polarization of M2 macrophages through different pathways. Exosomes from colorectal cancer cells are found to have high levels of miR-934, which can reduce PTEN expression. While downregulation of PTEN expression induces the PI3K/Akt pathway and promotes subsequent M2 polarization. Similarly, exosomal miR-934 has been found to promote M2 polarization of macrophages [92]. As reported before, miR-145 can induce the polarization of M2 macrophages in colorectal cancer cells. Cancer cells that secrete miR-145 in extracellular vesicles are related to the promotion of M2 macrophages by inhibiting enzymes that remove acetyl groups from histones, including histone deacetylase 11 (HDAC11) [93]. miR-145 can also enable communication between Macrophages and cancer cells, inducing tumor-promoting TME formation.

miRNAs can also inhibit tumor growth by inhibiting M2 polarization. IL-4 is an important promoter of M2 polarization in macrophages [94]. miR-195-5p inhibits IL-4-mediated M2 macrophage polarization by binding to the IL-4 3′-UTR, impairing the epithelial-mesenchymal transition (EMT) in colorectal cancer cells, thereby reducing Notch2 expression [95]. miR-224 was found to be involved in suppressing prostate cancer progression by downregulating the expression of Tribbles homolog 1 (TRIB1). TRIB1-induced M2-like macrophage polarization is inhibited by IKB-zeta in prostate cancer. Thus, the downregulation of TRIB1 expression by miR-224 suppressed M2 macrophage polarization in the TME [96].

M1 macrophages are the dominant phenotype in normal immune responses and can produce proinflammatory cytokines with a tumor cell- and microbe-killing activity and participation in TH1 (type I T helper cell) immunity [10]. miRNAs can also regulate M1 macrophage polarization in different cancers. miR-155 was found to be involved in driving M1 polarization in macrophages. Exosome-mediated expression of miR-155 and miR-125b-2 has been reported to be involved in the repolarization of M1 macrophages in pancreatic cancer cells [97,98]. miR-142-3p is commonly overexpressed in TME and can promote M1 macrophage planning and M2 macrophage apoptosis, thereby inhibiting tumor cell growth [99]. M2 macrophages overexpressing miR-142 selectively modulate TGF-β receptor-1 and can trigger apoptosis in M2 macrophages [99]. miR-99b is another promoter of M1 macrophage polarization. Restoring miR-99b expression can inhibit the m-TOR and κB-Ras2 molecular pathways to induce M1 polarization of macrophages and enhance antitumor immunity by enhancing the ability of macrophages in phagocytosis and antigen presentation [100].

In addition, several miRNAs were found to have dual roles in tumor suppressor and tumor promotion and could promote the polarization of M1 or M2 macrophages under different conditions. miR-200c mediates M2 polarization of macrophages by enhancing PAI-22 expression and promoting IL-10 secretion to enhance breast cancer progression [101]. miR-200c also can induce M1 polarization of macrophages through enhancing levels of GM-CSF and inhibiting breast cancer progression [102]. Similarly, miR-125a and miR-125b are reported to promote the M1 macrophage polarization, but also promote the anti-inflammatory M2 polarization. A significant increase in the M1/M2 ratio could be found using macrophages transfected with nanoparticles containing miR-125b. Mediated by activation of TLR-2 or TLR-4 and downstream myeloid differentiation factor (MyD88), M2 macrophages are found to have higher levels of miR-125a-5p than those in M1 macrophages. [103]. miR-125a-5p targets the transcription factor Kruppel-like factor 13 (KLF13), and its downregulation reduced the LPS-induced M1 phenotype and enhanced the IL-4-induced M2 polarization [104].

### 3.3. Exosomes Derived miRNAs in the Interaction of TAMs and Cancer Cells

Exosomes are important mediators of intercellular communication under pathological and physiological conditions [105]. Recently, exosome-derived miRNAs have been found to mediate cellular communication within the TME and, to varying degrees, immunomodulation [106]. miRNA transfer mediated by exosomes is an important method of communication between tumor cells and macrophages. miRNAs in exosomes from macrophages have been shown to lead to the progression and metastasis of cancer cells [107]. Through exosome transfer, miR-223 can be transferred from IL-4-activated macrophages to breast cancer cells and promote tumor cell proliferation [108,109]. In hepatocellular carcinoma TME, miR125a/b contained in exosomes released by TAMs affect the tumor cell division process and stem cell nature by targeting CD90. [110]. M2 macrophages can produce a variety of exosomal miRNAs to exert their immunosuppressive and tumor-promoting effects. miR-21-5p and miR-155-5p were found to be enriched in M2 macrophage-derived exosomes and were able to downregulate Brahma-related gene-1 (BRG1) expression to activate the Wnt/β-catenin pathway to promote colon cancer metastasis [111]. Enriched levels of miR-95 in M2 macrophage-derived exosomes were shown to induce prostate cancer aggressiveness and EMT, leading to poor prognosis and pathological characteristics [112]. miR-130b-3p in M2 macrophage-derived extracellular vesicles was found to promote cancer cell proliferation, migration, invasion, and angiogenesis in gastric cancer cells [113]. Furthermore, exosomes containing miR-487a secreted by M2 macrophages were effectively taken up by gastric tumor cells in the TME, and downregulation of TIA1 expression then promoted tumor progression [114]. miR-155-5p in M2 macrophage-derived exosomes was found to be significantly associated with increased IL-6 expression, which was capable of mediating tumor immune escape through IL-6-related pathways [115].

Macrophage-derived exosomal miRNAs were found to have important effects on the development or maintenance of tumor cell therapy resistance. miR-21 secreted by M2 macrophages prevented apoptosis and induced cisplatin resistance in tumor cells. This mainly relies on delivery to gastric cancer cells and targeting of PTEN to activate the PI3K/AKT pathway [116]. Under hypoxic conditions, abundant miR-223 could be found in macrophage-derived exosomes. Similarly, to miR-21, miR-223 can also translocate into cancer cells and induce resistance to cisplatin through the PTEN-PI3K/AKT signaling pathway. Lower miR-223 levels have been found to be associated with better prognosis [117]. Exosomal miR-365 from TAMs induces gemcitabine chemical blockade by activating cytidine deaminase in pancreatic cancer. Uptake of miR-365-containing exosomes by tumor cells confers gemcitabine resistance [118]. miRNAs, such as miR-22-3p, miR-27a-3p, and miR-221-3p, can be delivered to glioma stem cells via macrophage-derived extracellular vesicles, resulting in a poor tumor prognosis, mesenchymal phenotype, and resistance to radiation therapy [119]. Therefore, targeting these miRNAs or exosomes may be a promising approach to suppress tumor progression or therapy resistance.

Most published studies describe only one or more forms of exosomes for each type of cancer. However, exosomes in the TME contain many miRNAs that can inhibit tumors, rather than promote them. miR-142 and miR-223 are effectively transferred from macrophages to liver cancer cells by exosomes. These RNAs reduce the expression of report protein and endogenous protein one and insulin-like growth factor-1 receptor and inhibit cancer cell proliferation [120]. miR-125a and miR-125b have been found to regulate the expression of cancer stem cell marker CD90 to inhibit cancer cell proliferation [110].Tumor necrosis factor-like weak inducer of apoptosis (TWEAK) stimulated macrophages inhibit metastasis of epithelial ovarian cancer via exosomal shuttling of microRNAs [121]. In breast cancer, miR-130 is loaded into exosomes by triggering M1 polarization of macrophages to inhibit the growth and metastasis of breast cancer [122]. Therefore, we must fully recognize the communication role between the exogenous body in the cancer cells and the TME, which can inhibit the promotion pathway of cancer and enhance the cancer suppression pathway. This may help develop new ideas to improve tumor treatment pathways.

### 3.4. miRNAs Act as a Bridge for Cancer Cells to Affect the Function of TAMs

miRNAs secreted from cancer cells can affect immune cells in the TME, especially macrophages, and thus indirectly regulate tumor development. miRNAs carried in exosomes or EV released from cancer cells act mainly in the phenotypic switching of macrophages. As mentioned above, the M2 polarization of macrophages is beneficial to the proliferation and metastasis of tumor cells. Targeting tumor cell-derived exosomal miRNAs to induce polarization in macrophages can be an effective approach to controlling cancer. There are several examples. Exosomal miR-222-3p secreted by ovarian cancer cells can induce an M2-like phenotype by regulating the SOC3/STAT3 pathway, thereby creating an immunosuppressive TME [123]. Melanoma-derived exosomes can deliver miR-125b-5p, which leads to the polarization of M2 macrophages by targeting LIPA [104]. Tumor-derived exosomal miR-138-5p was able to regulate TAM polarization by inhibiting lysine demethylase 6B (KDM6B), suppressing the M1 phenotype, and promoting M2 macrophages [124]. miR-203 exosomes derived from colorectal cancer cells could help monocytes differentiate into macrophages and thus help cancer cells grow, proliferate, and migrate [125]. Under hypoxic conditions, miR-940 exosomes can be released from ovarian cancer cells to induce an M2-like phenotype in macrophages, thereby enhancing the aggressiveness of ovarian cancer [126]. Furthermore, under hypoxic conditions, miR-103a-loaded EVs transferred from lung cancer cells to macrophages could enrich M2-like macrophages [127]. Exosomes containing miR-let7a that were released from hypoxic cancer cells were likewise found to polarize the M2 phenotype of macrophages. In addition to hypoxia-related pathways, cancer cell-derived miRNAs can affect the function of macrophages through multiple pathways. Tumor-derived miR-let7a induces an M2-like phenotype in macrophages by regulating the insulin/AKT/m-TOR signaling pathway, thereby enhancing tumor aggressiveness [128]. Tumor cell-secreted miR-103a was able to downregulate PTEN expression in macrophages, which in turn activated the AKT/STAT3 pathway and promoted angiogenesis and immunosuppression [127]. The idea of targeting tumor-derived miRNAs to induce macrophages to polarize toward the M1 phenotype, thereby enhancing the antitumor effect, has also been experimentally validated. Several anticancer compounds have been shown to increase levels of miR-16 in exosomes from cancer cells, which, after being phagocytosed by M2 macrophages, induced their M1 polarization by inhibiting the NF-kB pathway [129]. Similarly, pancreatic cancer cells secreted exosomes carrying miR-155 and miR-125b-2 could promote M1-like phenotypic polarization of macrophages and are targets with therapeutic potential [130]. In conclusion, cancer cells can use miRNAs as a bridge to regulate key signaling pathways in macrophages and affect the biological functions of macrophages. Targeting this interaction between cancer cells and macrophages may yield new anticancer therapies.

**Figure 1:**
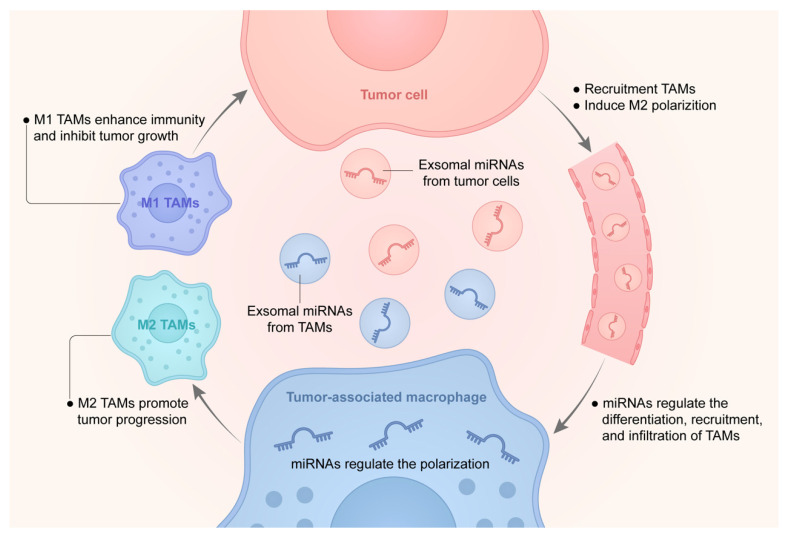
A graphic summary of the interactions among TAMs, tumor cells, and miRNAs.

In Table 1**,** we summarized reported microRNAs related to tumor-associated macrophages with function. In addition, we briefly illustrated the interactions among macrophages, tumor cells, and miRNAs in Figure 1.

## 4. Clinical Application and Prospect

Once the exact biological mechanism of miRNAs has been determined, these can then be therapeutically targeted. As anticancer therapeutics, miRNA-based therapies can reduce the level of oncogenic miRNAs or enhance the levels of tumor suppressor miRNAs [78]. Studies suggest that the efficacy and safety of miRNA-related treatments are superior to siRNA-based therapies [154]. Several studies of miRNA therapy have been reported. Intratumoral injection of miRNA-based drugs can promote their specificity and efficacy and decrease side effects. Intratumoral injection of cationic liposome/pVAX-miR-143 complex has been found to inhibit subcutaneous tumor growth in vivo [155]. This drug inhibited tumor progression in an early experimental lung cancer metastasis model following administration to the circulatory system. miR-19a-3p in macrophages can significantly downregulate the expression of Fos-associated antigen-1 (Fra-1). Intratumoral injection of miR-19a-3p can downregulate the expression of Fra-1 to reduce the invasion ability of breast cancer effectively [156].

TAM-secreted microvesicle delivery of miR-142-3p after propofol stimulation has been reported to inhibit tumor growth in tumor-bearing mice. Therefore, drug regulation of miRNA delivery in the TME has a potential anticancer value [157]. Exosomes derived from pancreatic cancer cells induce M1 macrophages to assume an M2 phenotype. However, this could be reversed by the administration of exosomes that had been transfected with miR-155 and miR-125b2, thereby returning M2 cells to the antitumor M1 phenotype [130]. Similarly, macrophages in glioblastoma TME secrete exosomes containing miR-21, levels of which are related to M2 polarization and temozolomide resistance. The application of pacritinib, a STAT3-related pathway inhibitor, reduces the secretion of miR-21-containing exosomes from macrophages to overcome temozolomide resistance in tumors [158].

Several clinical trials have recently reported the results of TargomiRs, which are minicells loaded with miR-16-based mimic miRNA targeted to EGFR [159]. Results of a first-in-human phase 1 clinical trial in malignant pleural mesothelioma showed acceptable safety and early activity of TargomiRs in patients, supporting further use of TargomiRs in combination with chemotherapy or immune checkpoint inhibitors. Locked nucleic acid (LNA)-based anti-miRNA drugs can work in different diseases. Cobomarsen (MRG-106) is an LNA-based drug targeting miR-155 to treat various hematological malignancies. The results of phase I clinical trials showed that MRG-106 was well tolerated by humans and had clinical activity [160]. Although both basic research and early clinical findings have shown the great potential of miRNA-mediated drugs, to date, no phase 3 clinical study results have been published, highlighting the challenges needed to successfully develop drugs.

miRNAs play an important role in the interaction of information between different types of cells in the TME, and aberrant expression of miRNAs has been shown to be a striking feature of many types of cancer. Several studies have explored the unique roles of miRNAs in cancer diagnosis, prognostic inference, and precision treatment evaluation. Upregulation of miR-195-5p can inhibit IL-4 secretion and M2-like TAM polarization [95]. In colorectal cancer patients, low levels of miR-195-5p were associated with shorter overall survival in colorectal cancer patients [161]. miR-130a can inhibit PPARγ protein expression and regulate macrophage polarization. Downregulated miR-130a expression was found to be a predictor of poor staging and prognosis in lung cancer [162]. In cases of diffuse large B-cell lymphoma, high expression of miR-21 was related to poor prognosis [163]. The level of miR-92a is negatively correlated with the infiltration level of macrophages in the TME and tumor stage and is an independent predictor associated with the prognosis of breast cancer patients [164]. A prospective phase II clinical trial reported that patients with low miR-31-3p expression showed improved overall treatment response rates compared with rates in patients with high miR-31-3p expression, as well as improved progression-free survival and overall survival [165]. miRNA expression profiling successfully classifies poorly differentiated tumors with higher accuracy than mRNA can. In breast cancer, miRNA expression profiles are found to be related to estrogen and progesterone receptor status, proliferation, and tumor stage. miRNAs can even be used to further precisely identify molecular subtypes of breast cancer and differentiate ductal carcinoma in situ from invasive ductal carcinoma [166,167]. In the case of leukemia, miRNAs can serve as early disease biomarkers and also differentiate between chronic and acute leukemia [168,169]. Research on miRNAs as biomarkers is still mostly at the laboratory stage and sometimes conflicting. Ultimately, a more comprehensive determination of the role of miRNAs in pathogenesis, disease progression, and response to therapy is urgently needed. Adequate research evidence is needed to find the optimal compromise between the sensitivity and specificity of biomarkers and the risk of obtaining false-negative results. Methodological issues and the lack of clear standardized criteria make it difficult to validate the reliability of miRNA biomarkers in clinical practice. In any case, considering the important role of miRNAs in the TME, miRNAs have shown substantial potential in predicting the outcome of chemotherapy, radiotherapy, or immunotherapy, and survival prognosis [78].

## 5. Conclusions

Cancer is a complex systemic disease, and the TME is an important “soil” for tumor occurrence, growth, and metastasis. In the TME, cancer cells interact with various types of cells. This complex communication network is strongly regulated and influenced by miRNAs. In the more than two decades since the first miRNAs were discovered, their biology has expanded considerably. More and more biological functions of miRNAs have been identified, and the number of therapeutic applications of miRNAs has increased significantly. These miRNAs may be identified as important regulators of immune processes, including macrophage polarization and macrophage–tumor cell communication, as well as therapeutic tools for various human cancers. 

As macrophages are one of the most abundant cells found in the TME, the role of miRNAs in regulating their biological functions was the focus of our review. The effect of macrophages on tumors is two-sided. M1 macrophages have antitumor activity, whereas M2 macrophages have oncogenic activity. Polarization of macrophages is an important process for macrophages to function. Macrophages also have additional phenotypes beyond the M1/M2 typing system. The role of miRNAs in regulating macrophage polarization has been intensively studied and can be divided into tumor-promoting and tumor-suppressing categories. Notably, miRNAs sometimes have both tumor-promoting and -suppressing functions. Therefore, after the exact role of each miRNA is determined, its therapeutic targeting should be assessed, and a series of clinical studies performed. In addition, both cancer cells and macrophages can secrete exosomes containing miRNAs, which are potential candidates for liquid biopsy for cancer diagnosis and prognosis. 

Overall, macrophages are important immune cells in the TME, and miRNAs regulate tumor cell proliferation, metastasis, and therapeutic response mainly by affecting the polarization of macrophages. This regulatory effect has been observed in various cancers. Targeting macrophages and macrophages-related miRNAs provides a new approach to tumor immunotherapy. 

Therefore, more research is needed to correctly evaluate new miRNAs and gain a deeper understanding of the biological mechanisms of the currently described miRNAs. These miRNAs may be identified as important regulators of immune processes, including macrophage polarization and potential molecular targets, and may contribute to therapeutic tools associated with oncogenic or tumor suppressor functions in various human cancers.

## Figures and Tables

**Table 1:** Summary of reported MicroRNAs related to tumor-associated macrophages.

MiRNAs Related to Macrophages	Function	Interactions between miRNAs and Macrophages	Ref.
**Main Tumor-promoters**			
**miR-21a**	Tumor-promoting	Inducing M2 polarization of macrophages	[116]
**miR-205**	Tumor-promoting	Recruiting macrophages, inducing inflammation in TME	[131]
**miR-125a-5p**	Tumor-promoting	Inducing M2 polarization of macrophages	[132]
**miR-200b**	Tumor-promoting	Triggering M2 polarization of macrophages	[133]
**miR-200c**	Tumor-promoting/suppressor	Inducing M1 or M2 polarization	[134]
**miR-467**	Tumor-promoting	Enhancing TAM infiltration in TME	[135]
**miR-223**	Tumor-promoting	Inducing IL-6 secretion by macrophages	[108]
**miR-138-5p**	Tumor-promoting	Inducing M2 polarization	[124]
**miR-145**	Tumor-promoting	Inducing M2 polarization	[136]
**miR-1305**	Tumor-promoting	Inducing M2 polarization	[137]
**miR-375**	Tumor-promoting	Facilitating macrophage recruitment, M2 polarization	[90]
**miR-934**	Tumor-promoting	Inducing M2 polarization	[138]
**miR-29a-3p**	Tumor-promoting	Inducing M2 polarization	[139]
**miR-222-3p**	Tumor-promoting	Inducing M2 polarization	[123]
**miR-940**	Tumor-promoting	Inducing M2 polarization	[126]
**miR-16**	Tumor-suppressor	Inducing M1 polarization	[140]
**miR-103a**	Tumor-promoting	Inducing M2 polarization	[127]
**miR-301a-3p**	Tumor-promoting	Enrichment of M2 macrophages	[141]
**miR-132**	Tumor-promoting	Inducing M2b polarization	[142]
**miR-1246**	Tumor-promoting	Reprograming to M2 polarization	[143]
**miR-148b**	Tumor-promoting	Inducing TAM infiltration in tumors	[144]
**Main Tumor-suppressors**			
**miR-498**	Tumor-suppressor	Preventing M2 polarization of macrophages	[145]
**miR-770**	Tumor-suppressor	Preventing M2 polarization of macrophages	[146]
**miR-125b**	Tumor-suppressor	Reducing number of Macrophages in TME, repolarization of M1 macrophages	[87,98]
**miR-33**	Tumor-suppressor	Inducing M1 polarization of macrophages	[147]
**miR-130**	Tumor-suppressor	Triggering M1 polarization of macrophages	[148]
**miR-23a**	Tumor-suppressor	Promoting M1 polarization and inhibits M2 polarization	[149]
**miR-155**	Tumor-suppressor	Reprogramming to an M1-like phenotype	[97]
**miR-142-3p**	Tumor-suppressor	Preventing M2 polarization of macrophages, triggering apoptosis in M2 macrophages	[150]
**let-7c**	Tumor-suppressor	Preventing M1 polarization and inducing M2 polarization of macrophages	[151]
**miR-26a**	Tumor-suppressor	suppress the recruitment of macrophages	[89]
**miR-195-5p**	Tumor-suppressor	Inhibits IL-4-mediated M2 polarization of macrophages	[95]
**miR-224**	Tumor-suppressor	suppressed M2 macrophage polarization in the TME	[96]
**miR-99b**	Tumor-suppressor	induce M1 polarization of macrophages and enhance antitumor immunity by enhancing the ability of macrophages in phagocytosis and antigen presentation	[152]
**miR-340-5p**	Tumor-suppressor	Downregulation of miR-340-5p promoted TAM recruitment and M2 polarization	[153]
